# Biological representation of chemicals using latent target interaction profile

**DOI:** 10.1186/s12859-019-3241-3

**Published:** 2019-12-20

**Authors:** Mohamed Ayed, Hansaim Lim, Lei Xie

**Affiliations:** 1 Ph.D. Program in Computer Science, The Graduate Center, The City University of New York, New York, NY USA; 2 Ph.D. Program in Biochemistry, The Graduate Center, The City University of New York, New York, NY USA; 3 Department of Computer Science, Hunter College, & The Graduate Center, The City University of New York, New York, NY USA

**Keywords:** Machine learning, Genome-wide target binding, Chemical embedding, Fingerprint, Bioactivity, LTIP

## Abstract

**Background:**

Computational prediction of a phenotypic response upon the chemical perturbation on a biological system plays an important role in drug discovery, and many other applications. Chemical fingerprints are a widely used feature to build machine learning models. However, the fingerprints that are derived from chemical structures ignore the biological context, thus, they suffer from several problems such as the activity cliff and curse of dimensionality. Fundamentally, the chemical modulation of biological activities is a multi-scale process. It is the genome-wide chemical-target interactions that modulate chemical phenotypic responses. Thus, the genome-scale chemical-target interaction profile will more directly correlate with in vitro and in vivo activities than the chemical structure. Nevertheless, the scope of direct application of the chemical-target interaction profile is limited due to the severe incompleteness, biasness, and noisiness of bioassay data.

**Results:**

To address the aforementioned problems, we developed a novel chemical representation method: Latent Target Interaction Profile (LTIP). LTIP embeds chemicals into a low dimensional continuous latent space that represents genome-scale chemical-target interactions. Subsequently LTIP can be used as a feature to build machine learning models. Using the drug sensitivity of cancer cell lines as a benchmark, we have shown that the LTIP robustly outperforms chemical fingerprints regardless of machine learning algorithms. Moreover, the LTIP is complementary with the chemical fingerprints. It is possible for us to combine LTIP with other fingerprints to further improve the performance of bioactivity prediction.

**Conclusions:**

Our results demonstrate the potential of LTIP in particular and multi-scale modeling in general in predictive modeling of chemical modulation of biological activities.

## Background

The tremendous advances in computational techniques have reflected extensively on drug discovery, toxicology, environmental science, and other scientific fields. Machine learning has reduced the time and cost of testing chemical compounds in vitro and in vivo by identifying chemical structures that will possibly modulate desired or unwanted biological activities. It is critical to select relevant molecular features of chemicals for training a machine learning model of bioactivities. Chemical fingerprints have provided an easy and quick method to represent the molecules as a vector of binary bits that denote the existence or absence of internal substructures or functional groups [1]. However, there is a fundamental gap between the chemical fingerprint and the bioactivity: the chemical space has activity cliffs where a small change in structure may lead to substantially different bioactivities [2], since the bioactivity depends on both the structure of molecule and its biological targets in a cellular context.

The chemical modulation of biological activity is a complex process [3]. It starts from the interaction of chemicals with genome-wide macromolecular targets in the cell. A chemical not only binds to its primary target (on-target) but also often interacts with unexpected off-targets [3]. Both on-targets and off-targets collectively mediate phenotypic responses through biological networks. Thus, the introduction of genome-wide target binding profile, in principle, will fill in the gap between the chemical structure and biological activity and potentially increase the power of predictive modeling of the bioactivity modulated by the chemical.

Many methods have developed which link drugs to their phenotypes [4–13]. In spite of their successes, all of these methods share several common limitations. First, only observed data, which are highly biased and incomplete, are considered in the modeling. Second, these methods can only be applied to existing drugs which have observed biological or clinical activities. Thus, these methods are incapable of predicting bioactivity of novel chemicals.

Intuitively, a chemical can be represented by its known target binding profile in the form of affinity fingerprints [14], which can be then used as a feature for machine learning. However, observed chemical-target interactions are highly noisy, sparse, and biased. Only a small portion of chemicals have the full spectrum of binding affinity to the same set of biological targets in the chemical genomics databases [15, 16]. It is infeasible to carry out multiple experimental bioassays for tens of millions of chemicals in the same condition in order to obtain the affinity fingerprint. Furthermore, many unknown off-targets that have never been tested experimentally can exist for a chemical, and they may play important roles in modulating the biological activity [3]. Thus, it is not trivial to handle a large number of missing values in the observed bioassay data. A large number of computational methods have been developed to predict genome-scale chemical-target binding profiles [17–24]. However, an issue in directly applying the observed or predicted target binding profile as the feature to machine learning is its high-dimensionality. It may have an adversarial impact on the performance of machine learning, especially when the number of samples is small.

**Fig. 1:**
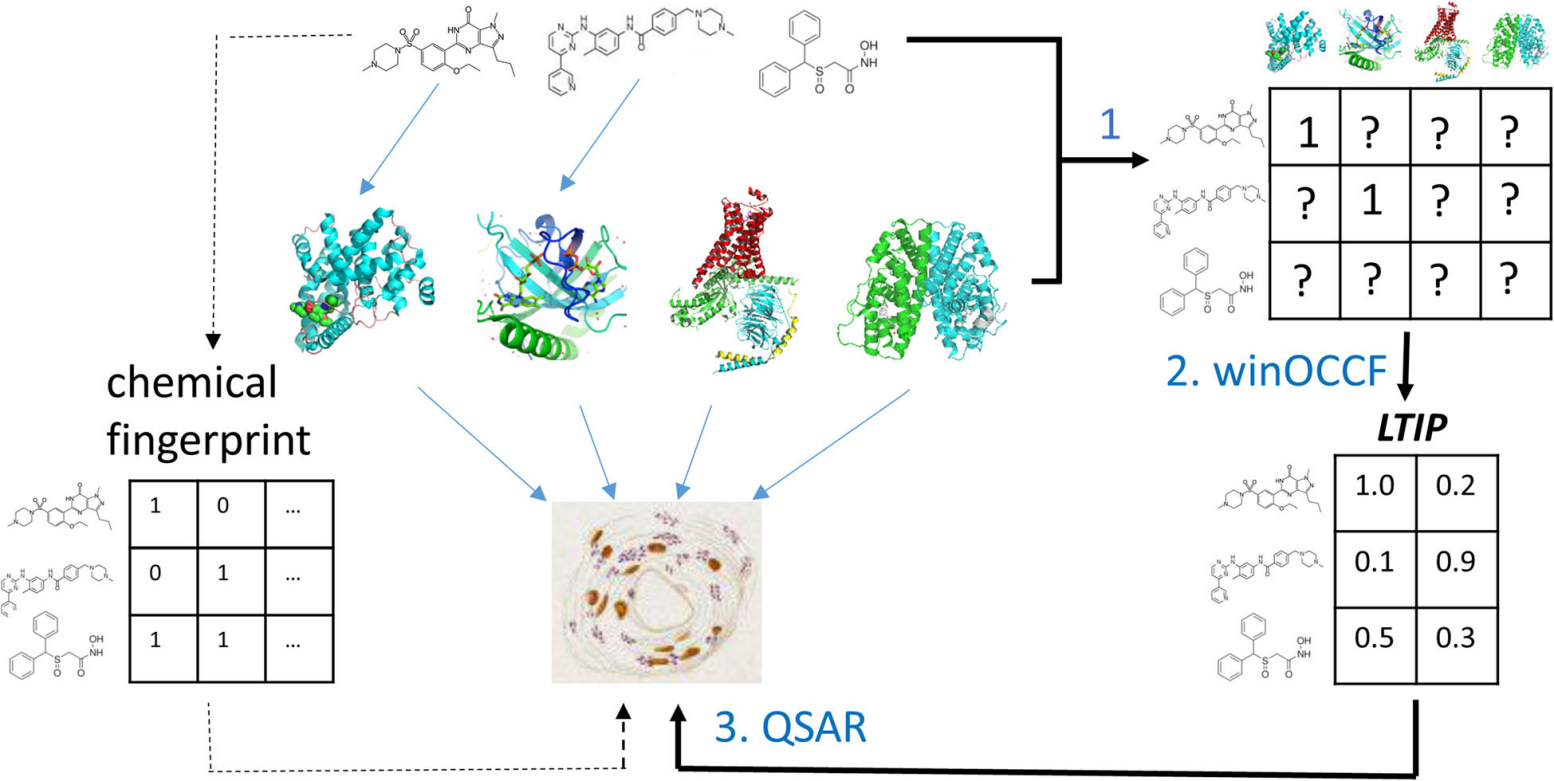
Schema of chemical fingerprint and proposed LTIP for Quantitative Structure-Activity Relationship (QSAR) modeling of bioactivity. Blue arrow represents observed chemical-protein interactions and protein-bioactivity associations. “1” and “?” denote observed and unknown interactions (i.e. missing data) in the interaction matrix, respectively

To address aforementioned challenges in the machine learning of the bioactivity modulated by novel chemicals, we propose to use a latent target interaction profile (LTIP) to represent the chemical, as shown in Fig. 1. The idea of LTIP is that the observed chemical activity profile can be embedded in a low-dimensional vector of hidden (latent) variables that can be considered as unobserved causal factors [25]. Previously we have developed novel methods REMAP [26] and its variations [27–29] that are based on a weighted imputed neighborhood-regularized One Class Collaborative Filtering (winOCCF) algorithm to predict geneome-wide drug-target interactions and to embed chemicals and target spaces into low-rank models. In the benchmark studies, REMAP can accurately reconstruct genome-scale chemical-target interactions. The top prediction from REMAP has been experimentally validated [29]. In this paper, we will use the low-rank model of chemicals derived from similar winOCCF algorithm as the representation of chemicals, i.e. LTIP. We compare LTIP with several conventional molecular fingerprints. Using cancer cell drug sensitivity as a benchmark, our results clearly shows that LTIP outperforms molecular fingerprints in many cases. Thus, LTIP provides a new tool for Quantitative Structure-Activity Relationship (QSAR) Modeling.

## Method

### Overview of the methodology

The fundamental idea of LTIP method is to represent chemicals in their biological context, in this case, as the low-dimensional representation (i.e. latent factor) of their global target binding profile. When representing the chemical-protein interactions as a bipartite graph where chemicals and proteins are nodes, and their interactions are edges, the LTIP is the chemical node embedding of the graph. There are basically three steps, as shown in Fig. 1.

#### Step 1

Given a set of chemicals of interest, the set is incorporated into a high-confident chemical-protein interaction matrix derived from ChEMBL [16], and Drugbank [30], which includes 199,338 chemicals and 6277 proteins.

#### Step 2

Given the known protein-chemical interaction matrix from the step 1, a weighted imputed neighborhood-regularized One Class Collaborative Filtering (winOCCF) algorithm is applied to derive the LTIPs of chemicals of interest.

#### Step 3

The LTIPs derived in Step 2 is used as features to train Quantitative Structure-Activity Relationship (QSAR) models from various machine learning algorithm.

### LTIP representation using winOCCF algorithm

The LTIP is constructed using a winOCCF algorithm [26]. The details of winOCCF have been published in ref. [26]. For the sake of completeness, a brief summary of winOCCF algorithm is given here.

Given *n* chemicals and *m* proteins, the inputs of winOCCF are the three matrices, *R*, *T* and *C*, which represent observed chemical-protein interactions, proteins-protein similarities, and chemical-chemical similarities, respectively. *R*(*i*, *j*) = 1 if the *i*^*th*^ chemical is associated with the *j*^*th*^ protein and *R*(*i*, *j*) = 0 otherwise. The *C*, *n* × *n* square matrix, will hold the similarity scores between chemicals such that 0 < *C*(*i*, *j*) < 1 will represent the Tanimoto coefficient-based similarity score between the *i*^*th*^ chemical and the *j*^*th*^ chemical. The protein matrix *T*_*m* × *m*_ will be in the same format where 0 < *T*(*i*, *j*) < 1 represent the protein sequence similarity.

The winOCCF optimizes the loss function in Eq. (1) to learn low-dimensional representations (i.e. low-rank features) of chemicals and proteins, *U* and *V*, respectively.

1 $$ {\displaystyle \begin{array}{l}\min \sum \limits_{\left(i,j\right)}{P}_{tw}\left(i,j\right){\left(R\left(i,j\right)+{P}_{imp}\kern0.28em \left(i,j\right)-{U}_{\left(i,\cdot \right)\cdot }{V^T}_{\left(j,\cdot \right)}\right)}^2\\ {}{+}_{Preg}\kern0.28em \left({\left\Vert U\right\Vert}^2+{\left\Vert V\right\Vert}^2\right){+}_{Pchem}\kern0.28em tr\left({U}^T\kern0.28em \left({D}_c-C\right)U\right)\\ {}{+}_{Pprot}\kern0.28em tr\kern0.28em \left({V}^T\kern0.28em \left({D}_T-T\right)V\right)\end{array}} $$

Where *P*_*wt*_(*i*, *j*) is the penalty of the loss. *P*_*wt*_(*i*, *j*) = 1.0 whenever *R*_*i*, *j*_ = 1 and otherwise *P*_*wt*_(*i*, *j*) ∈ [0, 1]. *P*_*imp*_(*i*, *j*) is the imputation of the unobserved association between chemical *i* and protein *j*. *P*_*imp*_(*i*, *j*) = 0 whenever *R*_*i*, *j*_ = 1 and otherwise *P*_*imp*_(*i*, *j*) ∈ [0, 1]. *p*_*reg*_ is the regularization parameter to control the regularization term $\left({\left\Vert U\right\Vert}_U^2+{\left\Vert V\right\Vert}_V^2\right)$ which is used to prevent overfitting. *D*_*c*_ and *D*_*T*_ is the degree matrix of *C* and *T*, respectively. The last two terms will force similar chemicals and proteins to have similar low-rank features. The detailed procedure of drug-target interaction network was presented elsewhere [26]. Briefly, the drug-target associations were obtained by integrating publicly available databases ChEMBL [16] (v23.1) and DrugBank [31] (v5.5.10). From ChEMBL, inhibition assays having *IC*_50_ ≤ 10 *μM* was regarded as active associations. Those with suboptimal confidence scores (i.e. confidence < 9) were excluded. From DrugBank, drug-target, drug-enzyme, drug-carrier, and drug-transporter associations were collected. MadFast software developed by ChemAxon (https://chemaxon.com/) was used to calculate chemical-chemical similarity matrix, and BLAST was used to calculate protein-protein similarity matrix. The integrated drug-target association network contains a total of 199,338 unique chemicals and 6277 unique proteins with 233,378 unique chemical-protein active pairs.

Given the low-rank factor of chemicals *U* that is optimized from the winOCCF, it can be used the features for various downstream predictive modeling tasks, e.g. *Y* ← *f*(*U*), where *Y* is the target variables, and *f*() is a mapping function. In our previously developed REMAP algorithm [26], the target variable is the genome-wide drug-target binding profile, and the mapping function is *U* × *V*. Different from original REMAP algorithm, the target variable is cancer cell line drug sensitivity, and the mapping function is a machine learning algorithm such as Random Forest. Because *U* can be considered as hidden variables that determine both observed and unobserved chemical binding profiles across all proteins, we term it as Ligand Target Interaction Profile (LTIP). In this study, the rank of *U* is set as 600. This rank is optimized for the drug-target interactions by REMAP [26].

### Performance evaluation

The main goal here is to evaluate whether or not LTIP has a better predictive power than standard chemical fingerprints. We did not compare LTIP with the experimentally determined affinity fingerprint, because they are not available for the chemicals in the most of cases, especially for novel chemicals, including our benchmark. In other words, affinity fingerprints are often not applicable in practice. Notably, we assume that all chemicals in our benchmark data set are novel, i.e. we do not know their molecular targets. We compared LTIP with standard fingerprints including Atom Pairs 2D (AP2D), Atom Pairs 2D count (AP2DC), Extended, Estate (ESFP), Klekota-Roth (KRFP), Klekota-Roth Count (KRC), MACCS, Pubchem (PCFP), and Substructure (SSFP).

In this study, cancer cell line drug sensitivity was used as a benchmark [32]. Seven cell lines that are across different tissue types including leukemia, prostate, lymphoma, glioma, endometrium, breast, and large intestine, and share the maximum number of chemicals assayed were selected. There are total 144 chemicals tested by all of 7 cell lines. Six state-of-the-art algorithms were used to build the machine learning models to assess the predictive power for standard fingerprints and LTIP. Particularly, we used support vector machines (SVR), XGboost (XGB), k-nearest neighbors regressor (KNR), Random Forests (RF), and Extra trees regressor (RF_EXTR). A necessary normalization procedure was applied to training and testing folds whenever it was needed. Although deep neural network has become the most popular method in machine learning, it may not be suitable for our data set since the number of features is usually more than 1000, but the total number of samples is only 144. The target variable is the area under of drug dose response curve (AUC) for each chemical against each cancer cell line, and is numerical. Thus a regression model was built using the fingerprint or LTIP of each chemical as the feature. It notes that the genomics features of the cell line were not used in model training, so we could directly compare the performance among different chemical representations. The data was randomly split into training/development and testing sets. The training/development set included 124 chemicals and was used for determining optimal hyperparameters using leave-one-out cross-validation (Additional file 1: Table S1-S5). After the hyperparameters were determined, an independent hold-out test set that included 20 chemicals was used to evaluate the performance of the trained model. The performance metric is Pearson’s correlation coefficient between actual and predicted AUC of anti-cancer drugs.

## Results

### LTIP is more accurate and robust than molecular fingerprints in predicting chemical anti-cancer activities

We have evaluated the performance of five of the state-of-the-art algorithms (RF, RF_EXTR, SVR, KNR, and XGB) with all possible combinations between seven cancer cell lines and fingerprints representations of the chemical compounds. The seven cancer cell lines are the large intestine, breast, endometrium, glioma, lymphoma, prostate, and leukemia. The chemical fingerprints that are compared with LTIP include Atom Pairs 2D (AP2D), Atom Pairs 2D count (AP2DC), Extended, Estate (ESFP), Klekota-Roth (KRFP), Klekota-Roth Count (KRC), MACCS, Pubchem (PCFP), and Substructure (SSFP).

**Fig. 2:**
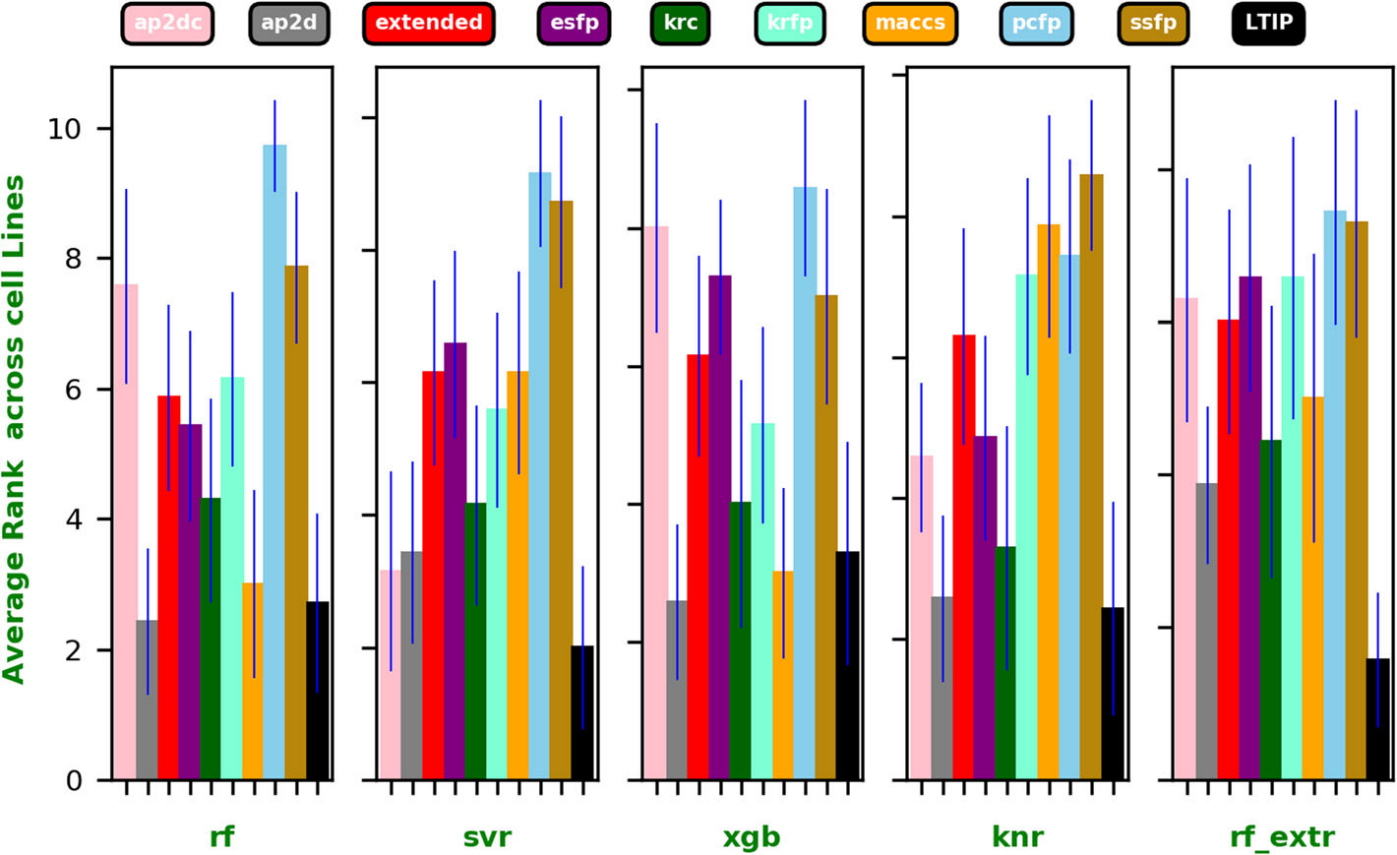
The average rank of Pearson’s correlation coefficients for each fingerprint and each algorithm across all 7 cell lines, respectively

**Fig. 3:**
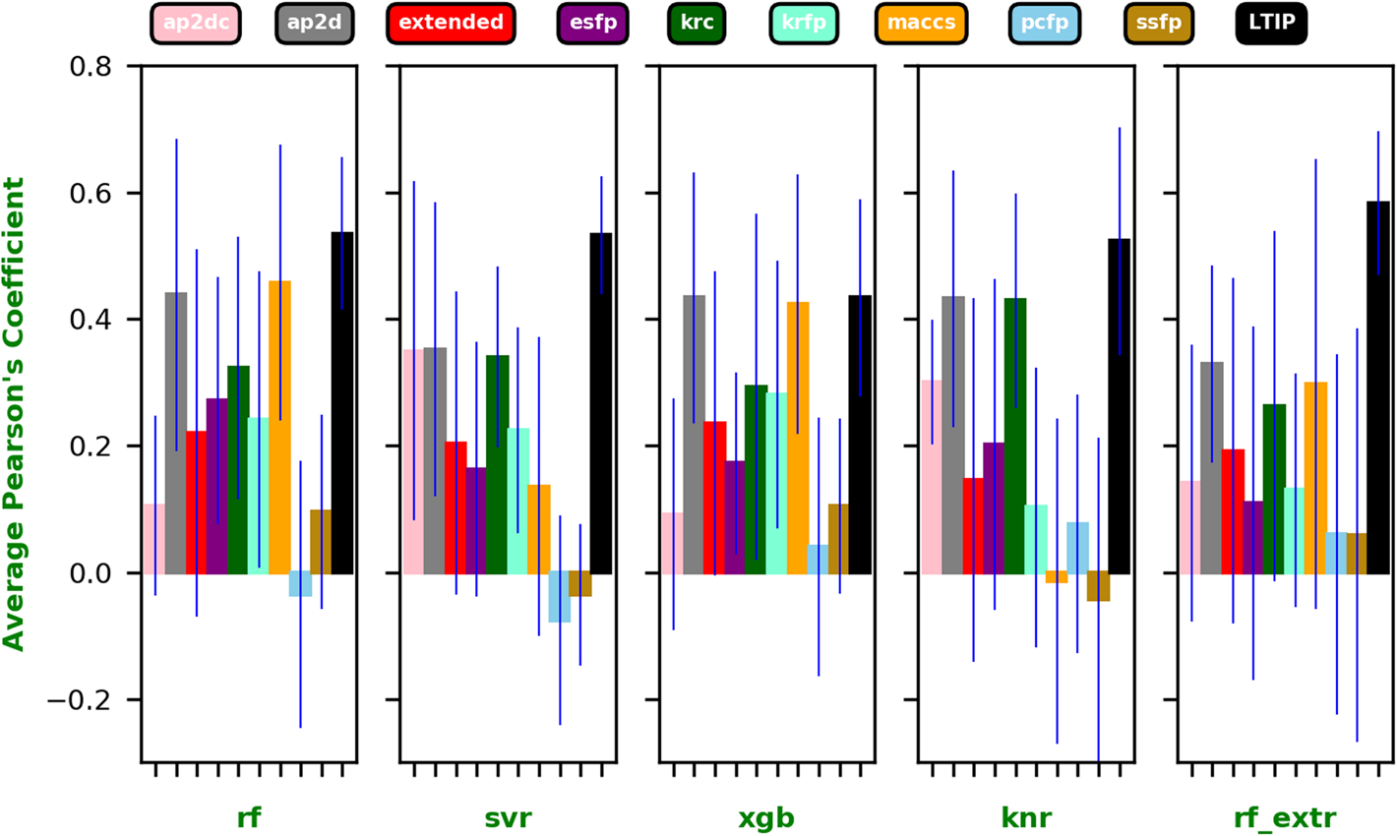
The average of the Pearson’s correlation coefficients for each fingerprint and each algorithm across all 7 cell lines

As shown in Figs. 2 and 3, the LTIP representation clearly outperformed all other fingerprints on an average regardless of the algorithm used. The best performance is achieved by LTIP trained with RF_EXRT with a Pearson’s Correlation Coefficient (PCC) of 0.58. It is about 75% higher than the second-best performance by the AP2D fingerprint (PCC = 0.33). Furthermore, the standard deviations of PCC of the LTIP is small across cell lines, compared with that of other fingerprints. Thus, performance of LTIP is robust. The robustness is a desirable property for a computational method in real-world applications. When the performance of different fingerprints is compared by the average ranking of PCC across cell lines, LTIP has the best rank when the QSAR model is built using SVR, KNN, and RF_EXRT.

### LTIP is complementary with other fingerprints

**Fig. 4:**
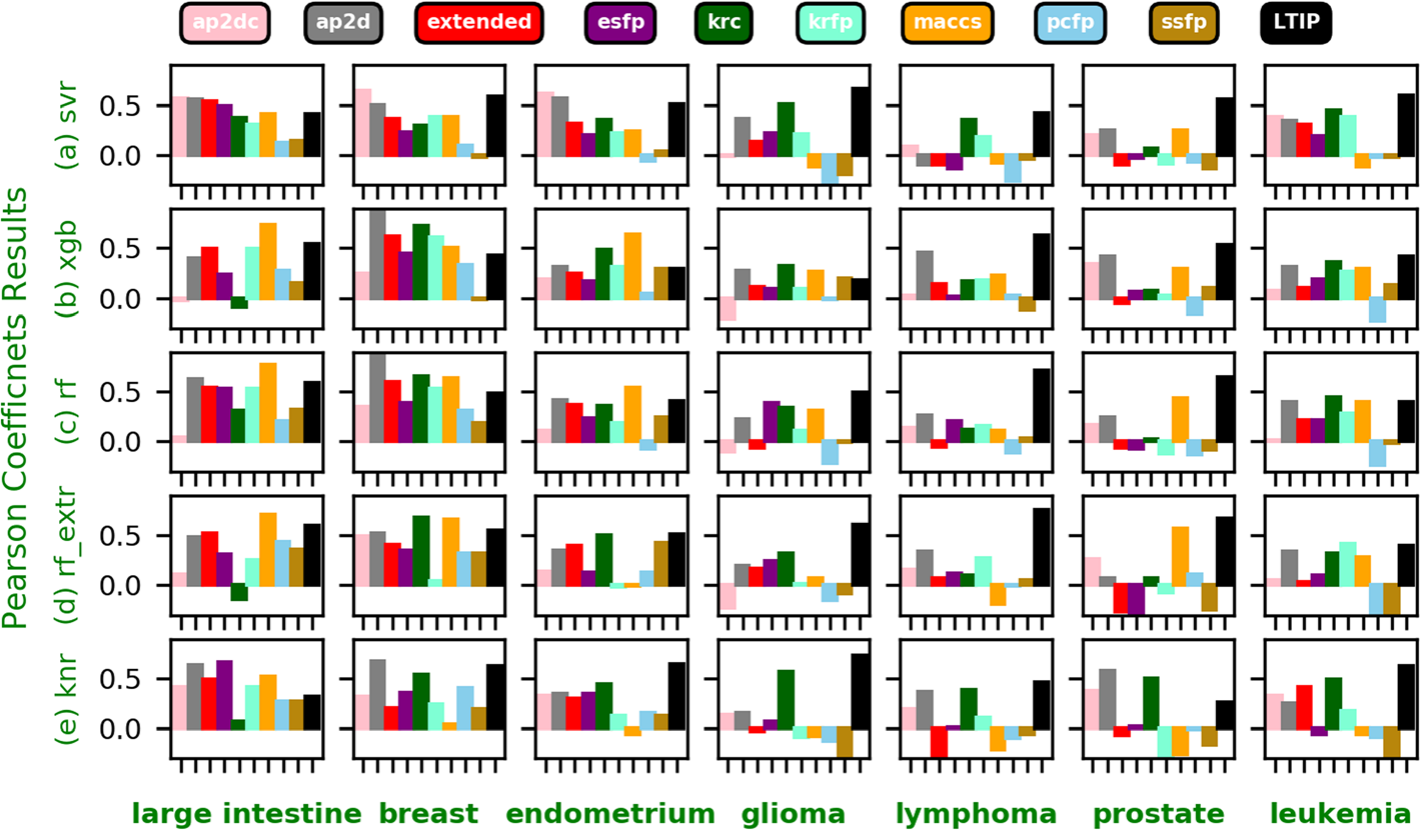
**a**-**e** Pearson’s correlation coeficients for each fingerprint, each algorithm, and each cell line

Although the performance of LTIP is more accurate and robust in general than the molecular fingerprint, the best performing fingerprint varies significantly for different cancer cell lines and algorithms, as shown in Fig. 4a–e. The results are summarized as follows.

#### Leukemia cell line

When applied to the Leukemia cell line, LTIP outperforms all other fingerprints across all algorithms except the RF_EXTR and RF algorithms. KRFP and KRC performs slightly better than the LTIP when using RF_EXTR and RF, respectively.

#### Prostate cancer cell line

The Prostate cancer cell line showed different results where LTIP significantly outperforms all other fingerprints using all algorithms except the KNR algorithm. When using the KNR algorithm, AP2D, AP2DC, and KRC outperform LTIP. Especially the AP2D followed by KRC outperforms the LTIP with a noticeable margin.

#### Lymphoma cell line

LTIP significantly outperforms other fingerprints when applied to the Lymphoma cancer line in all algorithms. The PCC of LTIP shows noticeable margins when compared with those of other fingerprints.

#### Glioma cell line

Although the SSFP, MACCS, KRC and AP2D fingerprints outperform LTIP when using the XGB algorithm, LTIP has shown comparable PCC. LTIP outperforms all fingerprints using the rest of the algorithms.

#### Endometrium cancer cell line

When applied to the Endometrium cancer cell line, the best performing fingerprints are AP2DC, KRFP, KRFP, LTIP, and LTIP for SVR, XGB, RF, RF_EXTR, and KNR, respectively.

#### Breast cancer cell line

LTIP fails to outperform all other fingerprints in all algorithms. The best performing fingerprints are AP2DC, AP2D, AP2D, KRC, and AP2D for SVR, XGB, RF, RF_EXTR, and KNR, respectively. It appears that atom pair-based fingerprints including AP2D and AP2DC is the winner.

#### Large intestine cancer cell line

LTIP fails to outperform all other fingerprints in all algorithms. However, not a single fingerprint dominates the best performer. The best performing fingerprints are AP2DC, MACCS, KRFP, KRFP, and ESFP for SVR, XGB, RF, RF_EXTR, and KNR, respectively.

**Fig. 5:**
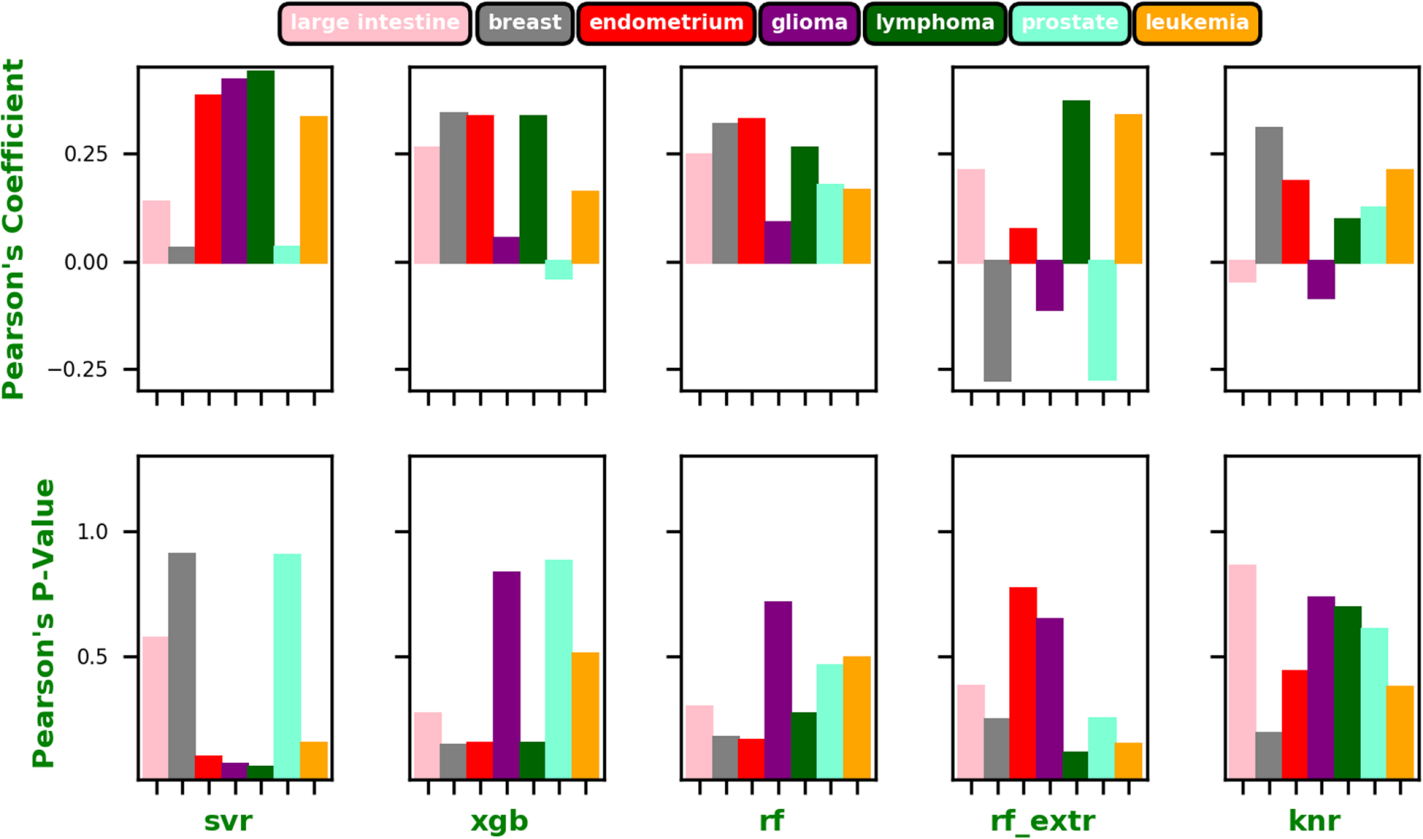
The Pearson’s correlation coefficients (top) and corresponding *p*-values (bottom) between the predicted AUCs obtained from the LTIP and those from KRFP

The correlations between the predictions using the LTIP feature and those using conventional fingerprints are weak, as shown in Fig. 5. The low R-squared values and high *P*-values (all > 0.05) suggest that LTIP is complement with chemical fingerprints.

Overall, LTIP is the best performed chemical represention for leukemia, prostate, lymphoma, glioma, and endometrium cell lines. AP2D and MCCS outperforms other fingerprints for breast and large intestine cell lines, respectively.

### Performance improvement by combining LTIP with molecular fingerprints

**Fig. 6:**
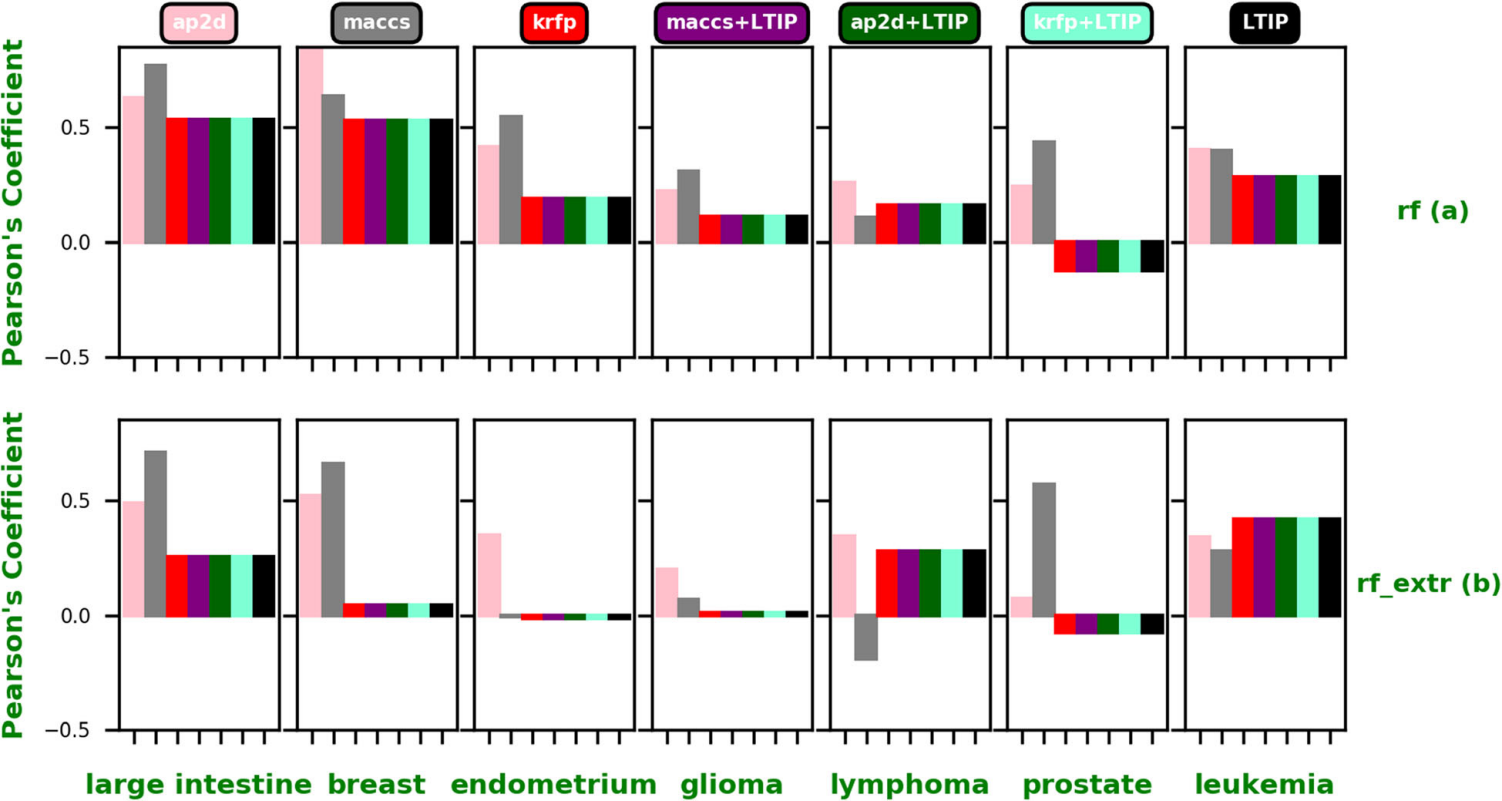
**a**-**b** The performance comparison of the concatenated features (AP2D + LTIP, MACCS+LTIP, and KRFP+LTIP) and individual fingerprints (MACCS, AP2D, KRFP, and LTIP) using **a** RF and **b** RF_EXTR algorithm, respectively

Since LTIP complements other fingerprints, it implies that combining LTIP with other fingerprints may improve the overall performance over individual ones. Therefore, we tested the performance of machine learning models by concatenating the LTIP feature with other fingerprint features. Specifically, we used the feature sets for AP2D, MACCS and KRFP fingerprints. New training sets are specified as AP2D + LTIP, MACCS+LTIP, and KRFP+LTIP. The same concatenation process was applied to the testing sets. The results varied across different kinds of concatenation. The results of comparing the concatenated (AP2D + LTIP, MACCS+LTIP, and KRFP+LTIP), non-concatenated fingerprints (MACCS, AP2D, KRFP) and LTIP are shown in Fig. 6a and b. Because of the high dimension of the concatenated feature vector, only RF and RF_EXTR were tested, and their results are summarized as follows.

#### Large intestine cancer cell line

When the RF_EXTR was used, AP2D + LTIP, and MACCS+LTIP outperformed LTIP with a noticeable margin. KRFP+LTIP showed comparable results to LTIP. All concatenated fingerprints outperformed the non- concatenated counterparts. The results with RF were a little different. All concatenated fingerprints outperformed the individual counterparts except MACCS that outperformed its concatenated counterpart (MACCS+LTIP).

#### Breast cancer cell line

Using the RF_EXTR algorithm, AP2D + LTIP outperformed all concatenated and individual fingerprints. All of the concatenated ones outperformed the individual fingerprints with a noticeable margin especially when KRFP+LTIP was used. When the RF algorithm was used, all concatenated fingerprints outperformed the non-concatenated counterparts except AP2D which outperformed its concatenated counterpart (AP2D + LTIP).

#### Endometrium cancer cell line

Using the RF_EXTR algorithm, MACCS+LTIP outperformed all concatenated and individual fingerprints with a noticeable margin. All of the concatenated ones outperformed the non-concatenated fingerprints with a noticeable margin especially KRFP+LTIP and AP2D + LTIP. Using the RF algorithm, MACCS+LTIP and AP2D + LTIP outperformed LTIP while KRFP+LTIP showed comparable results. MACCS clearly outperformed all concatenated and individual fingerprints with a noticeable margin.

#### Glioma cell line

Using RF_EXTR algorithm, all concatenated ones outperformed its individual counterparts and the LTIP. Using the RF algorithm, while MACCS+LTIP and AP2D + LTIP showed comparable results to each other, they outperformed all concatenated and individual fingerprints with a noticeable margin. All concatenated fingerprints outperformed the individual molecular fingerprints and the LTIP.

#### Lymphoma cell line

Using the RF_EXTR & RF algorithm, all concatenated fingerprints outperformed the individual molecular fingerprints and the LTIP.

#### Prostate cancer cell line

Using the RF_EXTR algorithm, AP2D + LTIP outperformed its counter individual fingerprint (AP2D) with a noticeable margin. Although KRFP+LTIP outperformed KRFP but it showed a low Pearson’s correlation coefficient. Using the RF algorithm, MACCS+LTIP has clearly outperformed all concatenated and individual molecular fingerprints as well as the LTIP with a noticeable margin.

#### Leukemia cell line

Using the RF_EXTR algorithm, MACCS+LTIP and AP2D + LTIP slightly performed better than their individual counterparts. Using the RF algorithm, all of the individual fingerprints has outperformed their concatenated counter parts.

In summary, the concatenation of LTIP with molecular fingerprints has improved its predictive performance in the most of cases when the RF_EXTR algorithm is used for the model training. It may be because that RF_EXTR performs better in handling extremely high-dimensional data than the RF.

## Discussion

In this proof-of-the-concept study, we have shown that LTIP is more accurate and robust in general than conventional chemical fingerprints in the predictive modeling of bioactivities regardless of the algorithm being used or the target being tested. There are several advantages of LTIP compared with the chemical fingerprints. First, LTIP embeds the genome-scale target binding profile, and thus, fills in one of the missing links between chemical structure and bioactivity. Second, the number of features in LTIP is low compared to most of the chemical fingerprints, thus, more robust machine learning models can be built. Third, the complementarity of LTIP suggests that LTIP combined with other fingerprints may improve the predictive performance for certain targets which is clearly seen for many of the cancer cell lines. Fourth, the high predictive power of LTIP suggests that it will present a better similarity measure between chemicals, which suffers from activity cliffs when the Tanimoto coefficient of chemical fingerprints is used.

Since the generalization power of machine learning algorithm is often sensitive to the nature of data, the optimal performance of fingerprints depends on the choice of machine learning method. Although LTIP shows promising results in most cases, it does not perform well in the combination with conventional fingerprints in one case, i.e. breast cancer cell line. The concatenation of two complementary fingerprints certainly encodes more information, but significantly increases the dimensionality of feature vector. In addition, due to the small sample size used in this study, the data in some cell lines may be not well distributed. These factors will impose difficulties in the learning. The performance of LTIP can be improved along several directions. Technically, the accuracy of LTIP depends on both the quality of chemical genomics data used and the underlying algorithms for learning latent factors. With the advance in high-throughput techniques, we expect a rapid increase in the coverage of chemical and genomic spaces. On the other hand, the winOCCF method used in the paper has limitations in representation learning, since it can only provide a linear mapping between the original feature space and the latent space. In this regard, deep learning could be a promising approach to the LTIP coupled with the increasingly availability of chemical genomics data. Fundamentally, even if LTIP can accurately encode the information of genome-wide target interaction profile, it may not completely fill in the gaps between chemical structure and bioactivity. The chemical-target interaction in vivo depends on the pharmacogenetics of individuals. For example, a single nucleotide polymorphism may significantly alter the chemical-target interaction or drug metabolism. Thus, the incorporation of genomics information could be critical for the embedding of chemicals in the context of precision medicine. Furthermore, the molecular targets (e.g. proteins) do not work alone but interact with each other. A gene interacting network view for the chemical representation may provide stronger correlations between the chemical structure and the bioactivity than LTIP. It will be interesting to use the same strategy for LTIP: to embed the biological pathway activity perturbed by chemicals into their latent space. From the point of view of model building, the complementary nature of LTIP with the chemical fingerprints suggests that other types of ensemble models in addition to the feature concatenation could improve the predictive modeling. The significant performance diversity from different learning algorithms and fingerprints implies that case-based reasoning could be an effective strategy to build the ensemble model [33].

## Conclusion

Although the chemical fingerprint has a long history in QSAR modeling and virtual screening, it has fundamental flaws in linking chemical structure with bioactivity, due to the hierarchy and modular nature of biological system. The direct use of molecule target binding profiles of chemicals may fill in the gap between the chemical structure and the bioactivity but faces problems of a large number of missing values and high dimensionality. In this proof-of-the-concept study, we demonstrate that the latent feature embedded from the sparse, noisy, and biased target binding profile could be a more accurate and robust molecular representation than the conventional fingerprints for the predictive modeling of bioactivity. In principle, the same concept can be extended to represent higher level perturbations by the chemical than the target binding, thus provide a general framework for the multi-scale modeling of chemical modulation of phenotypes. With the exponential increase of chemical genomics data and rapid advances in machine learning, we expect that the multi-scale chemical embedding of bioactivity could be a powerful tool in the predictive modeling of chemical modulation of bioactivity.

## Supplementary information

**Additional file 1:** **Table S1.** Hyperparameters used for SVR grid search. **Table S2.** Hyperparameters used for XGB grid search. **Table S3.** Hyperparameters used for RF grid search. **Table S4.** Hyperparameters used for RF_EXTR grid search. **Table S5.** Hyperparameters used for KNR grid search.

## Data Availability

The implementation of REMAP algorithm is available at https://github.com/hansaimlim/REMAP

## Abbreviations

AP2D: Atom Pairs 2D
AP2DC: Atom Pairs 2D count
AUC: Area under of drug dose response curve
ESFP: Estate fingerprint
KNR: k-nearest neighbors regressor
KRC: Klekota-Roth count
KRFP: Klekota-Roth fingerprint
LTIP: Latent target interaction profile
PCFP: Pubchem fingerprint
QSAR: Quantitative Structure-Activity Relationship
RF: Random Forest
RF_EXTR: Random Forest Extra Tees Regressor
SSFP: Substructure fingerprint
SVR: Support Vector Regressor
winOCCF: Weighted imputed neighborhood-regularized One Class Collaborative Filtering
XGB: XGboost
