# Supplementary material for: Biological representation of chemicals using latent target interaction profile
Source: BMC Bioinformatics. 2019 Dec 20;20(Suppl 24):674. doi: 10.1186/s12859-019-3241-3 (PMC6924142; doi:10.1186/s12859-019-3241-3)
Supplement: Supplementary file 1 — Additional file 1: Table S1. Hyperparameters used for SVR grid search. Table S2. Hyperparameters used for XGB grid search. Table S3. Hyperparameters used for RF grid search. Table S4. Hyperparameters used for RF_EXTR grid search. Table S5. Hyperparameters used for KNR grid search. [file 12859_2019_3241_MOESM1_ESM.pdf]

Supplemental Material

Table S1 Hyperparameters used for SVR grid search

SVR Hyperparameters name	Hyperparameters description	Value
Kernel	Type of Kernels used	{ 'linear', 'rbf','poly','sigmoid'}xx
C	The penalty parameter of the error term	{ 0.5,1,10,30}
gamma	The coefficient used with kernels ,' rbf' ,' sigmoid' and 'poly'	{'auto',0.0001, 0.0005, 0.001, 0.005, 0.01, 0.1}
coef0	Only used as independent term with kernels 'poly' and ' sigmoid'	{0.00,0.03,0.05}
degree	The polynomial kernel,' poly' , function degree	{3,5,7}
epsilon	The distance between the predicted and actual value within which no penalty is associated with the training loss	{0.1,0.2,0.3}

Table S2. Hyperparameters used for XGB grid search

XGB Hyperparameters name	Hyperparameters description	Value
**reg_alpha**	L1 regularization term that will control weights. The higher the value the mode conservative the model	{0, 0.1, 0.2, 0.3}
**reg_lambda**	L2 regularization term that will control weights. The higher the value the mode conservative the model	{0, 0.1, 0.2, 0.3}
**max_depth**	The maximum depth of the model’s built tree. The deeper the tree, the more likely the model will suffer from overfitting	{ 35 }
**n_estimators**	The number of boosted trees that will be built and fit.	{50, 100, 200, 1000}
**objective**	The learning task of the XGBRegressor() object instance. ’reg:linear’ means linear regression	{’reg:linear’}
**learning_rate**	It is used to control overfitting. It makes the boosting more conservative by reducing the features’ importance.	{0.1, 0.2, 0.3, 0.4}

Table S3. Hyperparameters used for RF grid search

RF Hyperparameters name	Hyperparameters description	Value
**min_impurity_split**	The threshold above which split will happen while growing the tree. It is also called early stopping threshold. This is to prevent overfitting.	{1e-7, 1e-6, 1e-5}
**min_samples_split**	The minimum number of samples required at an internal node for a split to happen. This is to prevent overfitting.	{2, 5, 7, 18}
**min_samples_leaf**	The minimum number of samples that must exist at each leaf.	{1, 3, 5, 7, 18}
**n_estimators**	The number of trees in the forest.	{1000}
**max_features**	The number of features to consider when looking for the best split	{10, 20, 30, 40, 50, 60, 70, 80, 90}

I.

Table S4. Hyperparameters used for RF_EXTR grid search

RF_EXTR Hyperparameters name	Hyperparameters description	Value
**min_impurity_split**	The threshold above which split will happen while growing the tree. It is also called early stopping threshold. This is to prevent overfitting.	{1e-7, 1e-6, 1e-5, 1e18}
**min_samples_split**	The minimum number of samples required at an internal node for a split to happen. This is to prevent overfitting.	{2, 5, 7, 18}
**min_samples_leaf**	The minimum number of samples that must exist at each leaf.	{1, 3, 5, 7, 18}
**n_estimators**	The number of trees in the forest.	{1000}
**max_features**	The number of features to consider when looking for the best split	{10, 20, 30, 40, 50, 60, 70, 80, 90}

Table S5. Hyperparameters used for KNR grid search

KNR Hyperparameters name	Hyperparameters description	Value
weights	The neighbor’ s function that are being used. ‘ uniform’ uses an equal weight for each neighbor. ’ distance’ will use the inverse of the distance so a closer neighbor will have a higher weight and a further neighbor will have a lower weight.	{’uniform’,’distance’}
n_neighbors	The number of nearest neighbors that will be used to find the final prediction.	{3,5,18}
algorithm	The algorithm used to find the nearest neighbor. ‘auto’ will try to find the best algorithm to find the nearest neighbor based on the samples passed when fitting the model. available algorithms are, ’ ball_tree’ , kd_tree’ and ‘brute’	{ ‘auto’ }
n_jobs	The number of parallel jobs while searching for the nearest neighbor.	{18}
